# Selective denervation for cervical dystonia

**DOI:** 10.3171/2022.9.FOCVID2291

**Published:** 2023-01-01

**Authors:** Megan M. J. Bauman, Nikita Lakomkin, Robert J. Spinner

**Affiliations:** Department of Neurologic Surgery, Mayo Clinic, Rochester, Minnesota

**Keywords:** cervical dystonia, spasmodic torticollis, selective denervation, modified Bertrand, peripheral nerve

## Abstract

Cervical dystonia (spasmodic torticollis) is a condition that involves sustained, involuntary contraction of neck and shoulder muscles, leading to abnormal movements and head posture. The authors present the case of a 41-year-old man with severe right rotational torticollis for 1.5 years due to predominant right cervical paraspinal and left sternocleidomastoid muscle hyperactivity. Following failed medical management, the patient elected to undergo surgical treatment for his torticollis. In their video, the authors discuss the steps of selective denervation using a modified Bertrand procedure, highlighting the associated anatomy and surgical planes. At the 1.5-year follow-up, the patient had no pain and his head position remained straight.

The video can be found here: https://stream.cadmore.media/r10.3171/2022.9.FOCVID2291

## Transcript

In this operative video, we highlight the anatomy and operative steps of a selective denervation procedure for the treatment of cervical dystonia.

### 0:30 History.

The patient is a 41-year-old man with a 1.5-year history of spontaneously head turning to the right. Initially, the patient was able to voluntarily correct his head turning. However, over the course of time, his symptoms became more prominent, and he developed a continuous involuntary pulling sensation that would result in his head turning to the right. This sensation eventually became painful and uncomfortable. Further, while the patient was able to modulate the head turning with sensory cues, Botox and Botox variants unfortunately failed to provide any symptomatic relief.^1,2^

### 1:10 Diagnosis and Patient Imaging.

The patient was diagnosed with cervical dystonia, also known as spasmodic torticollis, by a movement disorder neurologist. On physical examination, he demonstrated a right rotational torticollis of more than 60°. Additionally, his special movement disorder study showed increased activity in the right cervical paraspinal muscles and the left sternocleidomastoid muscle.

### 1:38 Modified Bertrand.

Given the patient’s worsening symptomatology, the patient consented to having a selective denervation procedure performed in the seated position. Compared to the standard Bertrand procedure, our modified technique omits identification of C1 branch.^3^ Eliminating the C1 nerve transection decreases operative time by not having to identify the variable anatomy of C1 and minimizes risk to the vertebral artery.^4–6^ We expose the posterior element of C1 and perform a myotomy of right-sided rectus capitis posterior major and inferior oblique muscles. We also perform myectomy of the semispinalis capitis and/or splenius capitis muscles, all of which are supplying branches of C2 and C3. We also denervate the contralateral sternocleidomastoid through a small oblique incision.

### 2:40 Selective Denervation of Right Cervical Paraspinal Muscles Slide.

The procedure occurs in two stages, with the first being selective denervation of the right cervical paraspinal muscles. To access the plane of denervation and myotomy/myectomy, four muscles must first be appreciated and identified. Trapezius is the superficial-most muscle upon our opening. Deep to the trapezius muscle lies the splenius capitis, followed by the semispinalis capitis muscle. Finally, semispinalis cervicis is present as the deepest muscle among those mentioned. Similar to opening individual chapters of a book, we deepen the dissection and identify the plane between the semispinalis capitis and semispinalis cervicis muscles.

### 3:47 Midline Posterior Cervical Skin Incision.

A posterior midline incision is used from the occiput to C7.

### 4:14 Developing Plane Between Right Semispinalis Capitis and Cervicis Muscles.

Upon establishing our plane between semispinalis capitis and semispinalis cervicis, we now have access to rectus capitis posterior major and inferior oblique muscles, and the medial branches of C2–6 dorsal rami.

### 4:42 Division of Rectus Capitis Posterior Major and Inferior Oblique Muscles.

Myotomy of rectus capitis posterior major and inferior oblique muscles allows exposure to C1 posterior element and proximal exposure of C2 nerve, which is then divided.

### 5:36 Division of Medial Branches of Dorsal Rami.

The medial branches of C3 through C6 dorsal rami are traced to their respective foramina and divided.

### 5:51 Resection of Semispinalis Capitis Muscle.

Myectomy of the semispinalis capitis and/or splenius capitis muscle is performed.

### 6:10 Identification and Division of Lateral Branches of Dorsal Rami.

Further dissection deep to the semispinalis capitis in the gutter lateral to the facet joint allows exposure of these lateral branches of dorsal rami, which are then divided.

### 6:27 Selective Denervation and Division of Left Sternocleidomastoid Muscle.

The second stage of the procedure involves selective denervation and division of the left sternocleidomastoid muscle. For this part of the procedure, we perform a sternocleidomastoid myotomy in addition to neurectomy of the spinal accessory nerve branch supplying the sternocleidomastoid muscle, taking care to avoid the spinal accessory nerve branch supplying the trapezius.

### 6:57 Lateral Neck Skin Incision.

A 3-cm oblique incision is planned halfway between the pinna of the ear and the turn of the trapezius using one of Langer’s lines.

### 7:12 Identification of Great Auricular Nerve.

The great auricular nerve is identified, mobilized, and protected.

### 7:22 Division of Sternocleidomastoid Muscle.

Dissection 2 cm posterior to it, the sternocleidomastoid is divided moving from superficial to deep.

### 7:35 Identification of Spinal Accessory Nerve Branches to Sternocleidomastoid and Trapezius.

During this dissection, the spinal accessory nerve is identified, including its major branch to the trapezius and the several branches to the sternomastoid.

### 7:53 Division of Deep Head of Sternocleidomastoid Muscle.

Division of the sternocleidomastoid muscle is then completed, including the deep belly.

### 8:26 Stimulation of Branches Prior to Selective Denervation.

Use of a disposable nerve stimulator helps confirm the accessory nerve branches.

### 8:36 Denervation of Nerve Branch to Sternocleidomastoid and Maintenance of Branch to Trapezius and Completion of Muscle Division.

The branches to the sternocleidomastoid are divided and the branch to the trapezius is maintained.

### 8:52 Postoperative Course.

The patient’s intraoperative and postoperative course were uncomplicated. Postoperatively, he had no pulling sensation, and his head was straight. Presently, at 1.5-year follow-up, the patient continues to do exceedingly well. His head position remains straight, and he is able to turn it both ways with ease.

## Acknowledgments

We thank Caitlin M. Morris, Stephen P. Graepel, and Donna T. DeSmet.

## Disclosures

The authors report no conflict of interest concerning the materials or methods used in this study or the findings specified in this publication.

## Author Contributions

Primary surgeon: Spinner. Assistant surgeon: Lakomkin. Editing and drafting the video and abstract: all authors. Critically revising the work: all authors. Reviewed submitted version of the work: all authors. Approved the final version of the work on behalf of all authors: Spinner. Supervision: Spinner.

## Supplemental Information

### Patient Informed Consent

The necessary patient informed consent was obtained in this study.
